# Supplementary material for: Multi‐dataset identification of innovative feature genes and molecular mechanisms in keratoconus
Source: J Cell Mol Med. 2024 Sep 19;28(18):e70079. doi: 10.1111/jcmm.70079 (PMC11412914; doi:10.1111/jcmm.70079)
Supplement: Supplementary file 2 — Material S2. [file JCMM-28-e70079-s002.zip › 7.GSEA analysis/6.SNORA74B/KEGG_INTESTINAL_IMMUNE_NETWORK_FOR_IGA_PRODUCTION.html]

Details for gene set KEGG\_INTESTINAL\_IMMUNE\_NETWORK\_FOR\_IGA\_PRODUCTION[GSEA]

|  || Dataset | SNORA74B.group.cls#h\_versus\_l |
| Phenotype | group.cls#h\_versus\_l |
| Upregulated in class | l |
| GeneSet | KEGG\_INTESTINAL\_IMMUNE\_NETWORK\_FOR\_IGA\_PRODUCTION |
| Enrichment Score (ES) | -0.5403474 |
| Normalized Enrichment Score (NES) | -1.4367216 |
| Nominal p-value | 0.043564357 |
| FDR q-value | 0.30163682 |
| FWER p-Value | 0.867 |
Table: GSEA Results Summary

  

Fig 1: Enrichment plot: KEGG\_INTESTINAL\_IMMUNE\_NETWORK\_FOR\_IGA\_PRODUCTION      
 Profile of the Running ES Score & Positions of GeneSet Members on the Rank Ordered List

  

| SYMBOL | TITLE | RANK IN GENE LIST | RANK METRIC SCORE | RUNNING ES | CORE ENRICHMENT || 1 | CD86 | na | 436 | 0.628 | 0.0436 | No |
| 2 | TNFRSF13C | na | 3685 | 0.271 | -0.0258 | No |
| 3 | CCR10 | na | 4891 | 0.229 | -0.0401 | No |
| 4 | TNFSF13B | na | 6053 | 0.191 | -0.0565 | No |
| 5 | ICOSLG | na | 6672 | 0.171 | -0.0590 | No |
| 6 | ITGA4 | na | 7435 | 0.150 | -0.0675 | No |
| 7 | ITGB7 | na | 7862 | 0.139 | -0.0673 | No |
| 8 | TNFRSF13B | na | 9844 | 0.096 | -0.1159 | No |
| 9 | CD28 | na | 11178 | 0.072 | -0.1479 | No |
| 10 | HLA-DRB5 | na | 11281 | 0.070 | -0.1446 | No |
| 11 | HLA-DQB1 | na | 13160 | 0.045 | -0.1947 | No |
| 12 | AICDA | na | 14603 | 0.029 | -0.2337 | No |
| 13 | HLA-DOA | na | 16250 | 0.012 | -0.2801 | No |
| 14 | ICOS | na | 17341 | 0.001 | -0.3114 | No |
| 15 | IL15RA | na | 18009 | -0.006 | -0.3301 | No |
| 16 | HLA-DRB1 | na | 19037 | -0.017 | -0.3581 | No |
| 17 | HLA-DMA | na | 19878 | -0.027 | -0.3799 | No |
| 18 | CCL27 | na | 21088 | -0.041 | -0.4111 | No |
| 19 | IL5 | na | 23454 | -0.072 | -0.4729 | No |
| 20 | LTBR | na | 23537 | -0.073 | -0.4687 | No |
| 21 | HLA-DOB | na | 23582 | -0.074 | -0.4633 | No |
| 22 | CXCL12 | na | 23758 | -0.077 | -0.4614 | No |
| 23 | HLA-DRB4 | na | 24359 | -0.087 | -0.4710 | No |
| 24 | HLA-DRB3 | na | 24377 | -0.087 | -0.4637 | No |
| 25 | TNFSF13 | na | 25693 | -0.111 | -0.4917 | No |
| 26 | CD80 | na | 26418 | -0.126 | -0.5013 | No |
| 27 | TNFRSF17 | na | 27772 | -0.159 | -0.5261 | Yes |
| 28 | IL2 | na | 27917 | -0.162 | -0.5158 | Yes |
| 29 | CCL28 | na | 28142 | -0.169 | -0.5071 | Yes |
| 30 | IL10 | na | 28370 | -0.176 | -0.4980 | Yes |
| 31 | CCR9 | na | 29240 | -0.202 | -0.5050 | Yes |
| 32 | TGFB1 | na | 30144 | -0.231 | -0.5103 | Yes |
| 33 | CD40LG | na | 30278 | -0.236 | -0.4931 | Yes |
| 34 | HLA-DRA | na | 30472 | -0.244 | -0.4768 | Yes |
| 35 | HLA-DPA1 | na | 30909 | -0.263 | -0.4658 | Yes |
| 36 | MAP3K14 | na | 31321 | -0.285 | -0.4522 | Yes |
| 37 | IL6 | na | 32098 | -0.328 | -0.4452 | Yes |
| 38 | HLA-DMB | na | 32287 | -0.339 | -0.4203 | Yes |
| 39 | CD40 | na | 32420 | -0.349 | -0.3929 | Yes |
| 40 | IL4 | na | 32876 | -0.383 | -0.3717 | Yes |
| 41 | IL15 | na | 32921 | -0.387 | -0.3383 | Yes |
| 42 | CXCR4 | na | 33720 | -0.481 | -0.3183 | Yes |
| 43 | CCL25 | na | 34109 | -0.559 | -0.2795 | Yes |
| 44 | HLA-DPB1 | na | 34158 | -0.570 | -0.2299 | Yes |
| 45 | MADCAM1 | na | 34188 | -0.578 | -0.1790 | Yes |
| 46 | PIGR | na | 34346 | -0.631 | -0.1271 | Yes |
| 47 | HLA-DQA2 | na | 34566 | -0.745 | -0.0668 | Yes |
| 48 | HLA-DQA1 | na | 34604 | -0.798 | 0.0036 | Yes |
Table: GSEA details [plain text format]

  

Fig 2: KEGG\_INTESTINAL\_IMMUNE\_NETWORK\_FOR\_IGA\_PRODUCTION      
 Blue-Pink O' Gram in the Space of the Analyzed GeneSet

  

Fig 3: KEGG\_INTESTINAL\_IMMUNE\_NETWORK\_FOR\_IGA\_PRODUCTION: Random ES distribution      
 Gene set null distribution of ES for **KEGG\_INTESTINAL\_IMMUNE\_NETWORK\_FOR\_IGA\_PRODUCTION**
